# The debate on antifibrinolytics in liver transplantation: always, never, or sometimes?

**DOI:** 10.1016/j.bjane.2024.844562

**Published:** 2024-09-26

**Authors:** Eduarda S. Martinelli, Stuart A. McCluskey, Keyvan Karkouti, Carla A. Luzzi, Matthanja Bieze, Luiz Marcelo S. Malbouisson, André P. Schmidt

**Affiliations:** a University Health Network, Toronto General Hospital, Department of Anesthesia and Pain Management, Toronto, Canada; b University of Toronto, Temerty Faculty of Medicine, Department of Anesthesiology and Pain Medicine, Toronto, Canada; c Santa Casa de Porto Alegre, Serviço de Anestesia, Porto Alegre, RS, Brazil; d Faculdade de Medicina da Universidade de São Paulo (FMUSP), Programa de Pós-graduação em Anestesiologia, Ciências Cirúrgicas e Medicina Perioperatória, São Paulo, SP, Brazil; e University Health Network, Women's College Hospital, Sinai Health System, Department of Anesthesia and Pain Management, Toronto, Canada; f University Health Network, Peter Munk Cardiac Centre, Toronto, Canada; g Universidade de São Paulo, Hospital das Clínicas, Divisão de Anestesiologia, São Paulo, SP, Brazil; h Hospital de Clínicas de Porto Alegre (HCPA), Serviço de Anestesia e Medicina Perioperatória, Porto Alegre, RS, Brazil; i Hospital Nossa Senhora da Conceição, Serviço de Anestesia, Porto Alegre, RS, Brazil; j Universidade Federal do Rio Grande do Sul (UFRGS), Programa de Pós-graduação em Ciências Pneumológicas, Porto Alegre, RS, Brazil; k Universidade Federal do Rio Grande do Sul (UFRGS), Programa de Pós-graduação em Ciências Cirúrgicas, Porto Alegre, RS, Brazil

Over the past two decades, there has been a notable reduction in blood loss and blood product requirements during orthotopic liver transplantation (OLT).^1^ This improvement can be attributed to advancements in surgical techniques, faster laboratory processing times, new technologies, and therapies that allow for more rapid diagnosis and treatment of developing coagulopathies.^2^ One area that remains contradictory is the role of antifibrinolytic drugs in liver transplantation.

Despite the progress noted above, OLT is still associated with substantial blood loss and blood product transfusions, such that liver transplant recipients have the greatest requirement for blood products compared to any other solid organ transplant. The etiology of bleeding during OLT is multifactorial, with hyperfibrinolysis identified as a significant contributor to nonsurgical bleeding.^3^

Hyperfibrinolysis is a state of increased clot resolution that can lead to severe, potentially life-threatening bleeding, thereby increasing the risk of morbidity and mortality following liver transplantation.^3^ During the anhepatic phase of surgery, there is an increase in the concentration of tissue-type plasminogen activator, which can lead to increased plasmin formation and fibrinolysis, which is often exacerbated immediately after graft reperfusion.^3^ Plasmin can cleave both fibrin and fibrinogen, and the resulting cleavage products inhibit the cross-linking of fibrin, aggravating this fibrinolytic effect.^4^

Clinical diagnosis of hyperfibrinolysis has been challenging because it cannot be detected with standard coagulation assays (PT, PTT, INR). However, automated point-of-care viscoelastic tests (VET), such as thromboelastography (TEG) and rotational thromboelastometry (ROTEM), can accurately detect hyperfibrinolysis by measuring clot lysis over time in whole blood^2^. The limitations of these assays include requiring at least 20 minutes to detect hyperfibrinolysis, and the potential for false negative results in the setting of high plasmin-antiplasmin levels or low α_2_-antiplasmin levels,^2^ which can be present during OLT.

Antifibrinolytic drugs, which have been available since the 1960s, fall into two classes: the nonspecific serine protease inhibitor aprotinin, and the plasminogen activation inhibitors ε-aminocaproic acid (EACA) and tranexamic acid (TXA).^5^ Aprotinin is highly effective but has a less favorable risk-benefit profile due to its association with an increased risk of renal dysfunction and thromboembolic events.^6^ EACA and TXA likely have similar efficacy,^7^^,^^8^ but due to its more complicated renal clearance mechanism and higher cost, interest in EACA has decreased, and it has largely been replaced by TXA.^9^ Accordingly, TXA is recommended by studies for the prevention and treatment of hemorrhages due to primary and secondary hyperfibrinolysis in major surgeries,^10^ and, in 2011, the World Health Organization (WHO) added TXA to its list of essential medicines. The strong recommendation of the guidelines for managing severe perioperative bleeding has been consistent over the years in the use of TXA in various surgical contexts, including liver transplant, coronary artery bypass graft surgery, gynecological cancer surgery, obstetric bleeding, trauma, and major orthopedic procedures, showing that TXA can be beneficial both when administered therapeutically and, at times, prophylactically.^9^^,^^11^

When used prophylactically, TXA's efficacy is proven in various types of surgeries, such as trauma,^12^ postpartum hemorrhage,^13^^,^^14^ cardiac,^15^^,^^16^ orthopedics,^17^^,^^18^ and other non-cardiac procedures,^19^^,^^20^ but not yet in OLT surgeries. In some settings, such as trauma and postpartum hemorrhage, early use of TXA has been shown to also reduce mortality.^12^^,^^13^ In terms of safety, although most studies have found no increased risk of thromboembolic events with the prophylactic use of TXA, there are some indications that this may not be the case in all settings. In a randomized controlled trial in patients with acute gastrointestinal bleeding, the TXA group had a significantly higher incidence of venous thromboembolism than the placebo group.^21^ In another randomized controlled trial on non-transplant liver resections in a mostly non-cirrhotic population, the TXA group was more associated with major general complications, such as reoperation.^22^ The POISE-3 trial randomly assigned patients undergoing non-cardiac surgery to receive either 1 g of tranexamic acid or a placebo at the beginning and end of the procedure. The primary outcome was to assess the effect of tranexamic acid on bleeding 30 days after the surgery. Additionally, primary safety outcomes included myocardial injury after non-cardiac surgery, non-hemorrhagic stroke, peripheral arterial thrombosis, and venous thromboembolism, also assessed at 30 days. The results indicated that while tranexamic acid was beneficial for controlling bleeding, it was associated with a slightly increased risk of thrombotic complications.^19^

The therapeutic use of TXA in OLT procedures is supported by several clinical studies. Over the years, the European Society of Anaesthesiology Guideline has consistently emphasized the benefits of treating documented hyperfibrinolysis in liver transplantation surgeries.^9^^,^^11^ A meta-analysis of 69 randomized controlled trials indicated that TXA reduces red blood cell transfusion requirements compared to placebo without increasing the risk of deep vein thrombosis, pulmonary embolism, all-cause mortality, hospital length of stay, reoperation, myocardial infarction, stroke, or seizures. The limitation, however, was the high risk of bias related to the systematic overestimation of benefits.^23^ In a retrospective cohort study of 1799 patients, TXA effectively reduced red blood cell transfusion requirements without increasing the risk of thromboembolic events across a wide variety of liver transplant recipients, including those at low risk of bleeding or high risk of thromboembolic complications.^24^ A Cochrane review of 33 trials involving 1913 patients undergoing liver transplantation found no significant differences in 60-day mortality, rate of retransplantation, or increased incidence of thromboembolic episodes between TXA or control groups. Conclusions on outcomes, however, were based on only a few clinical trials, each with a high risk of random error due to the small number of patients.^25^ Another recent systematic review in liver transplantation patients also found that antifibrinolytic agents are unlikely to increase thrombotic complications or impair kidney function, though the authors suggested that further research is needed to clarify the optimal indications and dosages.^7^

The risk of thromboembolic events associated with TXA use is particularly concerning in OLT due to the heightened risk in cirrhotic patients, who often have a delicate balance of coagulation factors. End-stage liver disease (ESLD) disrupts coagulation homeostasis, leading to decreased levels of nearly all procoagulant factors, while liver-produced anticoagulants such as protein C, protein S, and antithrombin III are also significantly reduced. Concurrently, severe endothelial dysfunction from endotoxemia and nitric oxide dysregulation in ESLD patients causes increases in liver-independent coagulation factors like FVIII and vWF, and to a lesser extent, plasminogen activator inhibitor 1 (PAI-1). These changes likely create a new equilibrium between fibrinolytic and antifibrinolytic factors, making intraoperative management challenging.^26^ Thus, although there is no clear evidence indicating an increased risk of hypercoagulability from antifibrinolytics during liver transplantation, more robust studies, particularly randomized trials, are highly desirable to evaluate the feasibility of prophylactic TXA use in OLT procedures. Also, optimal dosing has yet to be established because the lowest effective dose of lysine analogs for patients with ESLD to treat hyperfibrinolysis is unknown. Currently, TXA is typically administered in either 1 to 2 g boluses or as an infusion of up to 30 mg.kg^−1^, while EACA, as a possible substitute, is given in doses ranging from 0.25 to 2 g, up to a maximum of 5 to 10 g.^26^

It is crucial to determine whether there is a specific population of liver transplant patients that would benefit the most from TXA without exceeding the potential risks. Patients with conditions such as renal failure or a prothrombotic state (including primary biliary cirrhosis, primary sclerosing cholangitis, hepatocellular carcinoma, portal vein thrombosis, and Budd-Chiari syndrome) may be at a heightened risk for thromboembolic complications. These individuals might not only derive less benefit from TXA but could also face a greater likelihood of adverse effects.^3^

The rapid recovery of hyperfibrinolysis following reperfusion in many patients raises questions about the necessity and impact of prophylactic TXA. Specifically, it is uncertain whether TXA would significantly alter the quantity of blood products required preceding the coagulation stabilization and, if there is a reduction in transfusion, would even translate to a reduction in mortality and morbidity.^27^ Additionally, the rapid return to normal fibrinolysis complicates the task of identifying which patients might benefit from TXA before the need becomes evident. Currently, prophylactic administration of antifibrinolytics for liver transplantation patients is not well supported, and it seems prudent to base treatment decisions on viscoelastic test results and to avoid the routine use of antifibrinolytics in these patients at this moment until further studies are concluded.^26^

In summary, understanding key aspects of perioperative coagulation in the context of OLT is essential to guide the use of antifibrinolytic agents, ultimately mitigating additional risks in this vulnerable population. Antifibrinolytic therapy should only be considered for liver transplantation recipients with significant bleeding when hyperfibrinolysis is either suspected or confirmed by VET.^26^ Sufficiently powered randomized controlled trials are needed to determine the effectiveness and safety of prophylactic tranexamic acid in the liver transplant population before it can be recommended for routine clinical use.

## Conflicts of interest

The authors declare no conflicts of interest.
